# Mössbauer Imaging

**DOI:** 10.6028/jres.092.032

**Published:** 1987-10-01

**Authors:** Stephen J. Norton

**Affiliations:** National Bureau of Standards, Gaithersburg, MD 20899

**Keywords:** image reconstruction, imaging, Mössbauer effect, Mössbauer imaging, Mössbauer tomography, tomographic image reconstruction, tomography

## Abstract

In a Mössbauer experiment, if a spatially-extended absorbing sample is rotated relative to a moving *γ*-ray source, lines of constant *γ*-ray Doppler shift are generated through the absorber parallel to the motion of the source. As a result, resonant absorption takes place along a series of parallel lines cutting through the absorber, where a particular line is determined by the velocity of the source. The result is a series of measurements of line integrals of the absorption coefficient through the absorber. An image or spatial map of the absorption coefficient distribution may then be reconstructed using tomographic image-reconstruction algorithms. Moreover, when measurements are recorded both as a function of the source velocity and the absorber rotational velocity, spectral information may also be recovered as a function of position. Spatial resolution is proportional to the rate of rotation of the absorber, but is ultimately signal-to-noise limited.

## 1. Introduction

**Figure 1:**
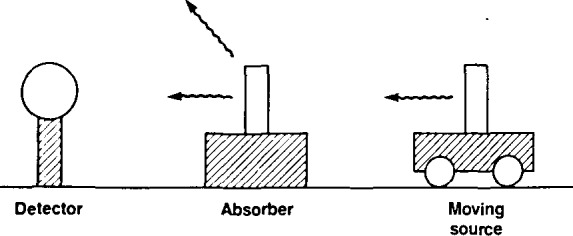
Conventional Mössbauer experimental arrangement with stationary absorber and moving source.

The Mössbauer effect is an important spectroscopic tool in materials science [1,2]^1^. In a typical Mössbauer experiment, *γ*-rays emitted by a radioactive source are allowed to impinge upon a sample of absorbing material; the transmitted or resonantly-scattered *γ*-rays are then subsequently detected (fig. 1). Thus, in a transmission experiment, a detector counts the number of *γ*-rays passing through the absorber, and in a scattering experiment the detector counts the *γ*-rays re-emitted after resonant absorption. The Mössbauer effect, or nuclear *γ*-ray fluorescence, is made possible by the recoilless emission and absorption of the *γ*-rays in nuclei embedded in a solid lattice. As a result, the resonant linewidths, relative to the *γ*-ray energy, are characteristically very narrow. Moreover, the resonant absorption can be “tuned” merely by moving the source at a small velocity relative to the absorber, which imparts some kinetic energy, or Doppler shift, to the *γ*-ray. Changes in source-absorber speeds as small as a few millimeters per second are often sufficient to destroy the resonance. In the conventional Mössbauerexperiment, an absorption spectrum of the material under study is generated by moving the source relative to the absorber and counting the transmitted *γ*-rays (or resonantly-scattered *γ*-rays in a scattering experiment) as a function of the relative source-absorber velocity.

In this paper, we propose the idea of Mössbauer imaging. An image is by definition some quantity or parameter of interest displayed as a function of position, i.e., a picture of the parameter as it is distributed in space. In Mössbauer imaging, the simplest example of such a parameter is the Mössbauerabsorption coefficient, and the aim would be to reconstruct and display its spatial distribution. A conventional Mössbauer experiment measures only a bulk average of the resonant absorption coefficient over the absorbing specimen. Thus, spatial inhomogeneities within an extended absorber, due either to variations in the absorption cross section of a pure material or to the mixture of different nuclei within a composite material, are inevitably averaged out in the bulk measurement process. A Mössbauerimaging experiment, however, would permit the reconstruction of a two-dimensional map of the Mössbauer absorption coefficient, that is, a two-dimensional picture of the strength of the resonant absorption as a function of position. In another, somewhat more complex, version of an imaging experiment, spectroscopic information as a function of position should also be recoverable, rather than merely differences in the absorption coefficient as a function of position. As a consequence, in the latter version, true Mössbauer spectroscopy can be performed in an imaging mode, so that heterogeneous or composite samples may be investigated.

The idea of Mössbauer imaging was inspired by the success of nuclear-magnetic-resonance (NMR) imaging, since NMR and the Mössbauer effect share some fundamental characteristics, both being nuclear resonance phenomena. NMR imaging has recently found notable success in diagnostic medicine [3]. While there are no foreseeable applications of Mössbauer imaging in medicine, applications to materials science are thought to exist. The ability to perform Mössbauer spectroscopy in an imaging mode, i.e., to do spectroscopy as a function of position within a sample, rather than in bulk, should prove to be of value in the analysis of heterogeneous materials. Although NMR and Mössbauerimaging may have different applications, it is instructive to compare the two techniques. In NMR imaging, resonant-frequency information is translated into spatial information by imposing a magnetic field gradient on a system of precessing nuclear spins. In Mössbauer imaging, we will see that by rotating the *γ*-ray absorber, a velocity gradient is imposed along the radial direction of the absorber and the resonance associated with a particular Doppler shift (analogous to a particular NMR precessional frequency) takes place along a family of lines perpendicular to the velocity gradient (analogous to the NMR magnetic field gradient). This process gives rise to a set of measurements of the line integrals of the absorption coefficient, from which an image can be recovered using well-known tomographic image reconstruction algorithms. These algorithms are mathematically similar to those employed in both x-ray and NMR tomography [4,5]. A more detailed description of the process by which the line-integral measurements arise is given in section 3.

The basis of the imaging approach can be most easily described using a classical interpretation of Mössbauer absorption, which will suffice for our purposes. As noted earlier, in this description the relative source-absorber velocity imparts a Doppler shift to the *γ*-ray. For purposes of illustration, consider the simplest case of a material with a single resonance line (neglecting isomer shifts); then resonance will take place at zero Doppler shift, or zero relative velocity between the source and absorber. By rotating an extended absorbing object relative to the source, lines of constant Doppler shift (or equivalently, of constant *γ*-ray energy) are generated across the absorber. As a result, resonant absorption takes place along one line at a time, giving rise to one line-integral measurement of the absorption coefficient. The location of the line is determined by three parameters: the absorber rotational velocity, the source velocity and the instantaneous position of the absorber during the measurement. From a complete set of such line- integral measurements, a spatial map of the *γ*-ray absorption can, in principle, be tomographically reconstructed. We shall see that the spatial resolution within the reconstructed image is proportional to the ratio of the natural linewidth of the resonance (in units of velocity) to the rate of rotation of the absorber, but is ultimately signal-to-noise limited. This means that, in principle, arbitrarily high resolution can be achieved, but at the expense of rapidly increasing detector integration times. In particular, the integration time will increase in direct proportion to the number of resolution elements (pixels) in the desired image. Thus, in a two-dimensional image, doubling the linear resolution will square the number of pixels, which in turn will square the required integration time. As a consequence, very high resolution will require exceedingly long integration times unless sources of high intensity become more readily available.

At this stage one might observe that the equivalent line integral measurements could be generated merely by directing a collimated beam of *γ*-rays at the absorber over a wide range of incident angles. This approach is analogous to conventional x-ray computerized tomography in a transmission experiment or to single photon emission tomography in a scattering experiment. The advantage of the proposed method is that an extended source can be used rather than a collimated source, which implies a substantially greater incident *γ*-ray flux and a corresponding enhancement in the signal-to-noise ratio.

In the following three sections, we consider the idealized case of the *γ*-rays incident parallel to the *x*-axis. This can be achieved by moving the source sufficiently far away so that the source subtends a small angle at the absorber. The assumption of parallel incidence simplifies the analysis considerably and permits an analytical solution to the image reconstruction problem; it also allows the analytical inversion of the complete Mössbauer spectrum at each point (section 3). Moreover, the parallel- incidence case also permits the closed-form derivation of the image point spread function, which is defined as an image of a point object (Dirac delta function) and characterizes the resolving power of the imaging technique (section 4). In section 5 we consider an extended source which gives rise to *γ*-rays incident over a range of directions that are no longer parallel. In the latter case, the image reconstruction cannot be inverted analytically as for parallel incidence, but the reconstruction can nonetheless be performed using iterative algebraic reconstruction techniques [4,5]. Although such iterative reconstruction methods often fail to provide the insight and intuition of analytical solutions (which permit, for example, the closed-form derivation of the image point spread function), such methods are often more flexible in incorporating *a priori* information into the inversion algorithm. An important example is the incorporation of *γ*-ray attenuation due to a variety of scattering and absorption (resonant and nonresonant) mechanisms. For relatively thick objects, attenuation can no longer be ignored in the inversion. For the sake of tractability, we will, however, ignore attenuation in the next several sections.

## 2. Standard Experiment

**Figure 2:**
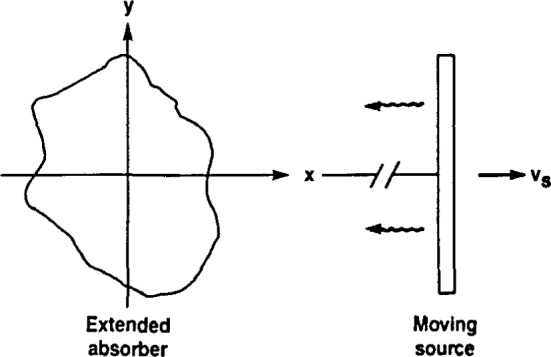
Two-dimensional absorber and distant moving source providing parallel *γ*-ray illumination.

Suppose a *γ*-ray emitted by a moving source is incident on a two-dimensional stationary absorber (fig. 2). For our purposes, it will suffice to describe the resonant absorption classically by modeling the nuclear resonance phenomenon, within a single absorbing nucleus, as a harmonic oscillator characterized by resonance frequency *ω*_a_ and linewidth *τ* (analogous to the mean lifetime of the *γ*-ray energy level) and driven by the electromagnetic field of the incident *γ*-ray. Letting *A*(*t*) denote the response of this elemental oscillator, we have

$$\ddot{A}(t)+\frac{1}{\tau }\dot{A}(t)+\omega_{a}^{2}A(t)=A_{\text{inc}}(t),$$ (1)

where

$$A_{\text{inc}}(t)=A_{0}\exp\left[i\omega_{0}\left(1-\frac{v_{s}}{c}\right)t\right]$$ (2)

is the *γ*-ray electromagnetic field. In eq (2)*v*_a_ is the velocity of the moving source, *c* is the velocity of light and *A*_0_ is a constant. Although one might also choose to include a finite linewidth in the *γ*-ray field *A*_inc_(*t*), we shall, for mathematical simplicity, lump all linewidth effects into the parameter *τ* in eq (1). Note that *ω*_0_*v*_a_/*c* in eq (2) is just the classical Doppler shift due to the motion of the source. In an equivalent notation, *ω*_0_*=E_0_/ħ* and γ*=ħ/τ*, where *E*_0_ is the energy of the incident *γ*-ray and γ is the (energy) linewidth. Now in eq (1), set

$$\omega_{a}=\omega_{0}\left(1-\frac{v_{a}}{c}\right),$$ (3)

where *v*_a_ is a parameter (in units of velocity) that allows for shifts in the nuclear resonance of a given nucleus. Thus, the “intrinsic velocity,” *v*_a_, is characteristic of different absorbing nuclei (or their environment). We will later allow *v*_a_ to vary with position. The solution of the differential eq (1) on substituting eq (2) is

$$A(t)=\frac{A_{0}e^{i\omega_{s}t}}{\omega_{a}^{2}-\omega_{s}^{2}+i\omega_{s}/\tau },$$ (4)

where

$$\omega_{s}\equiv \omega_{0}\left(1-\frac{v_{s}}{c}\right)$$ (5)

is the Doppler-shifted source frequency. Substituting eqs (3) and (5) into eq (4), and assuming *v*_s_ and *v*_a_< <*c*, eq (4) becomes

$$A(t)=\frac{A_{0}}{2\omega_{0}}\frac{e^{i\omega_{s}t}}{(v_{s}-v_{a})\omega_{0}/c+i/2\tau }.$$

Classically, the intensity of the re-emitted *γ*-ray is proportional to |*A*|^2^, i.e.,

$$I(v_{a}-v_{s})\equiv |A{|}^{2}={\left(\frac{A_{0}}{2\omega_{0}}\right)}^{2}\frac{1}{{\left(v_{a}-v_{s}\right)}^{2}{\left(\omega_{0}/c\right)}^{2}+{\left(1/2\tau \right)}^{2}},$$ (6)

or, in an equivalent notation,

$$I(v_{a}-v_{s})={\left(\frac{A_{0}}{2\omega_{0}}\right)}^{2}\frac{\hbar^{2}}{{\left(v_{a}/c-v_{s}/c\right)}^{2}E_{0}^{2}+\Gamma^{2}/4},$$

which is the familiar Lorentzian (or Breit-Wigner) distribution [1,2].

Because the detection of the re-emitted *γ*-ray is an incoherent process, we can regard eq (6) as proportional to the probability of the *γ*-ray re-emission as a function of the velocity difference *v*=*v*_a_−*v*_s_. To make eq (6) a valid probability density function in *v*, normalize eq (6) to have unit area when integrated with respect to *v*, i.e., define

$$P^{\left(\tau \right)}(v)\equiv \frac{2{\omega_{0}}^{3}}{\pi \tau A_{0}^{2}c}I(v)=\frac{v_{\tau }/\pi }{v^{2}+v_{\tau }^{2}},$$ (7)

where

$$v_{\tau }\equiv c/2\omega_{0}\tau$$

is the linewidth in units of velocity. From eq (7),

$$\int_{-\infty }^{\infty }P^{\left(\tau \right)}(v)dv=1.$$

One can also show that

$$\underset{v_{\tau }\to 0}{\lim}P^{\left(\tau \right)}(v)=\delta (v),$$ (8)

where δ(·) is the Dirac delta function.

Now let *σ*(*x, y, v*_a_) denote the resonant absorption cross section as a function of space and the intrinsic velocity *v*_a_. Then the expected number of counts, denoted by *f*^(^*^τ^*^)^(*v*_s_), is proportional to the function *σ(x, y, v*_a_) weighted by the probability density function *P*(*v*_a_−*v*_s_) and integrated over *v*_a_, and all space:

$$f^{\left(\tau \right)}(v_{s})=\int \int \int dxdydv_{a}\sigma (x,y,v_{a})P^{\left(\tau \right)}(v_{a}-v_{s}).$$ (9)

In this and subsequent integrals, we shall assume that the limits of integration are from − ∞ to + ∞ unless otherwise indicated. We can assume this by defining functions such as *σ*(*x, y, v*_a_ to be zero outside their support (i.e., beyond the boundaries of the absorber and the domain of *v*_a_, and so on). Now, for convenience, suppose we temporarily consider the limit of very small *v_τ_* (narrow resonant linewidth). As *v_τ_* goes to zero, the probability density function *P*^(*τ*)^(*v*_a_−*v*_s_) becomes highly peaked about *v*_a_=*v*_s_, and in the limit *v_τ_*→0, we can use eq (8) in (9). Thus, defining

$$f(v_{s})\equiv \underset{v_{\tau }\to 0}{\lim}f^{\left(\tau \right)}(v_{s}),$$

we have from eqs (8) and (9),

$$f(v_{s})=\int \int dxdy\sigma (x,y,v_{s}),$$

which is the expected result for the standard Mössbauer experiment, that is, *f*(*v*_s_) is the absorption cross section evaluated at *v*_a_=*v*_s_ averaged over the sample.

## 3. Imaging with Parallel *γ*-ray Incidence

**Figure 3:**
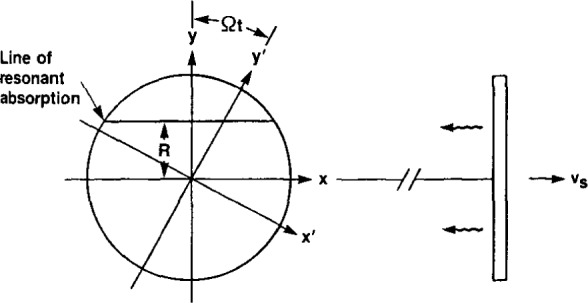
Rotating two-dimensional absorber with parallel *γ*-ray illumination. Resonant absorption takes place only along a line of “sensitivity” parallel to the *x*-axis at height *R*, where *R* is defined by the condition *R*Ω = *v*_s_−*v*_a_.

Now let the absorber rotate clockwise about the *z*-axis at constant angular velocity Ω. Let (*x, y*) denote a stationary coordinate system and let (*x′, y′*) denote a moving coordinate system embedded in the rotating absorber, as illustrated in figure 3. At *t*=0, let the stationary (*x, y*) and the rotating (*x′, y′*) coordinate systems coincide; at this instant denote the absorber’s cross-section function by *σ*_0_(*x, y, v*_a_). For *t*>0, the absorber has rotated through an angle Ω*t.* Define *σ_t_*(*x, y, v*_a_) as the absorption cross section relative to the stationary axes (*x*, *y*) at time *t;* then

$$\sigma_{t}(x,y,v_{a})=\sigma_{0}(x\prime ,y\prime ,v_{a}),$$ (10)

where

x′=xcosΩt−ysinΩt (11a)

y′=xsinΩt+ycosΩt, (11b)

or, inverting,

x=x′cosΩt+y′sinΩt (12a)

y=−x′sinΩt+y′cosΩt. (12b)

Again consider a *γ*-ray incident parallel to the *x*-axis from a source moving at velocity *v*_s_ in the positive *x*-direction (fig. 3). Resonant absorption will then take place when the total velocity *v*_a_+v_a_′ of a point on the absorber equals the source velocity *v*_s_, where *v*_a_ is the intrinsic material “velocity” and *v*_a_′ is the additional velocity contribution due to the motion of the absorber. Since the *γ*-ray is incident parallel to the *x*-axis, *v*_a_′ will be the *x*-component of the absorber velocity; thus, from eq (12),

$$v_{a}^{\prime }=\dot{x}=\Omega y.$$

Note that *v*_a_′ depends only on *y*, so that resonant absorption takes place along lines parallel to the *x*-axis. For the stationary absorber, the resonance condition was *v*_a_=*v*_s_; for the rotating absorber, the resonance condition is modified to read

$$v_{a}+\Omega y=v_{s}.$$ (13)

Thus, for specific values of Ω and *v*_s_, resonant absorption takes place along a line at height *y*=(*v*_s_−*v*_a_)/Ω parallel to the *x*-axis (fig. 3).

Now, in view of eq (13), for the rotating absorber, we can replace *v*_s_ in eq (9) by *v*_s_−Ω*y*, giving

$$\begin{array}{l} f_{t}^{\left(\tau \right)}(v_{s},\Omega )=\int \int dxdydv_{a}\sigma_{t}(x,y,v_{a})\\ \cdot P^{\left(\tau \right)}(v_{a}-v_{s}+\Omega y). \end{array}$$ (14)

Now considering a very narrow resonance, we again take the limit *v_τ_*→0. Defining

$$f_{t}(v_{s},\Omega )\equiv \underset{v_{\tau }\to 0}{\lim}f_{t}^{\left(\tau \right)}(v_{s},\Omega ),$$

and using, from eq (8),

$$\begin{array}{r} \underset{v_{\tau }\to 0}{\lim}P^{\left(\tau \right)}(v_{a}-v_{s}+\Omega y)=\delta (v_{a}-v_{s}+\Omega y)\\ =\frac{1}{\Omega }\delta [y-(v_{s}-v_{a})/\Omega ] \end{array}$$

in eq (14), we have

$$f_{t}(v_{s},\Omega )=\frac{1}{\Omega }\int \int dxdv_{a}\sigma_{t}[x,(v_{s}-v_{a})/\Omega ,v_{a}].$$ (15)

This is our fundamental equation from which we shall solve for the function *σ*_0_(*x′, y′, v*_a_) from the data *f_t_*(*v*_s_, Ω) recorded as a function of *v*_s_, Ω, and *t.* Below, we consider four special cases of increasing generality: 1) one-dimensional imaging with no *v*_a_ distribution (i.e., assuming a single spectral line at *v*_a_=0); 2) two-dimensional imaging with no *v*_a_ distribution; 3) one-dimensional image with an arbitrary and unknown *v*_a_ distribution; and finally 4) two-dimensional imaging with an arbitrary and unknown *v*_a_ distribution.

### One-Dimensional Imaging with No v_a_ Distribution

We consider the simplest imaging case here. Suppose the absorber is one-dimensional and rotating. For convenience, assume the absorber is aligned with the (rotating) *y*′-axis, and suppose further that the measurements are made at the instant the absorber rotates past vertical, i.e., when the rotating absorber coincides with the *y*-axis at *t* =0. We then have

$$\sigma_{t}(x,y,v_{a})=\sigma_{0}(y)\delta (x)\delta (v_{a}).$$

and eq (15) reduces to the simple result

$$f_{0}(v_{s},\Omega )=\frac{1}{\Omega }\sigma_{0}(v_{s}/\Omega ).$$

Thus, the spatial dependence of the one-dimensional cross section *σ*_0_(*v*) is given explicitly as a function of *v/*Ω.

### Two-Dimensional Imaging (Tomography) with No v_a_ Distribution

Here we let

$$\sigma_{t}(x,y,v_{a})=\sigma_{t}(x,y)\delta (v_{a}),$$ (16)

so that eq (15) becomes

$$f_{t}(v_{s},\Omega )=\frac{1}{\Omega }\int dx\sigma_{t}(x,v_{s}/\Omega ).$$ (17)

Recall that the measurements *f_t_*(*v*_s_, Ω) are made as a function of time as the absorber rotates. To make the *t*-dependence in eq (17) explicit, rewrite eq (17) as follows.

$$\begin{array}{l} f_{t}(v_{s},\Omega )=\frac{1}{\Omega }\int \int dxdy\sigma_{t}(x,y,)\delta (y-v_{s}/\Omega )\\ \;=\frac{1}{\Omega }\int \int dx\prime dy\prime \sigma_{0}(x\prime ,y\prime )\\ \;\cdot \delta (-x\prime \sin\;\Omega t+y\prime \cos\;\Omega t-v_{s}/\Omega ), \end{array}$$ (18)

where eqs (10) and (12b) were used in the last step. From eq (18)*f_t_*(*v*_s_, Ω) is a line integral through the two-dimensional function *σ*_0_(*x′, y′*) along a line parameterized by the radial distance *v*_s_/Ω and he angle Ω*t* from the *y*′-axis. The inversion of eq (18) for *σ*_0_(*x*′, *y*′) can be carried out by standard tomographic techniques. It is easiest to invert eq (18) using Fourier methods, as follows.

First define the one-dimensional Fourier transform of *f*_t_(*v*_s_, Ω) with respect to *v*_s_ as

$${\bar{f}}_{t}(k,\Omega )=\int dv_{s}f_{t}(v_{s},\Omega )\exp(-ikv_{s}).$$ (19)

Inserting eq (18) into (19) and interchanging orders of integration, gives

$$\begin{array}{l} {\bar{f}}_{t}(k,\Omega )=\int \int dx\prime dy\prime \sigma_{0}(x\prime ,y\prime )\\ \;\cdot \exp[ik\Omega (x\prime \sin\;\Omega t-y\prime \cos\;\Omega t)]. \end{array}$$ (20)

Now the two-dimensional Fourier transform, ${\tilde{\sigma }}_{0}(k_{x},k_{y})$, of *σ*_0_(*x′, y*′) is

$$\begin{array}{l} {\tilde{\sigma }}_{0}(k_{x},k_{y})=\int \int dx\prime dy\prime \sigma_{0}(x\prime ,y\prime )\\ \;\cdot \exp[-i(k_{x}x\prime +k_{y}y\prime )], \end{array}$$ (21)

and, on comparing eqs (21) and (20), we have

$${\tilde{f}}_{t}(k,\Omega )={\tilde{\sigma }}_{0}(-k\Omega \;\sin\;\Omega t,k\Omega \;\cos\;\Omega t).$$ (22)

Equation (22) is the well-known central-slice theorem in tomography [4,5]. In the present context it states that ${\tilde{f}}_{t}(k,\Omega )$, evaluated at time *t* and spatial frequency *k*, is the two-dimensional spatial Fourier transform, ${\tilde{\sigma }}_{0}(k_{x},k_{y})$, of *σ*_0_(*x′, y′*) evaluated on a line in the (*k_x_, k_y_*) Fourier plane through the origin at angle Ω*t* with the *k_x_*-axis. Thus, taking many revolutions of data and, during each revolution, stepping the value of *v*_s_ will generate sufficient data to provide complete coverage of the (*k_x_, k_y_*) Fourier plane, whereupon an inverse two-dimensional Fourier transform of ${\tilde{\sigma }}_{0}(x\prime ,y\prime )$yields the reconstruction *σ*_0_(*x′, y′*). Note that the required range of measured *v*_s_ values is from 0 to Ω*R*_max_, where *R*_max_ is the largest radial dimension of the absorber.

### One-Dimensional Imaging with Unknown v_a_ Distribution

In this case, let

$$\sigma_{t}(x,y,v_{a})=\sigma_{0}(y,v_{a})\delta (x).$$

For the one-dimensional absorber, again assume that the measurement is made as the absorber rotates past vertical at *t*=0. Then, substituting *σ_t_(x, y*, *γ*_a_) into eq (15) and setting *t* = 0, gives

$$f_{0}(v_{s},\Omega )=\frac{1}{\Omega }\int dv_{a}\sigma_{0}[(v_{s}-v_{a})/\Omega ,v_{a}].$$ (23)

We wish to solve for the two-dimensional function *σ*_0_(*y*, *v*_a_), which has one spatial dimension (*y*) and one spectral dimension (*v*_a_). Rewrite eq (23) as

$$f_{0}(v_{s},\Omega )=\frac{1}{\Omega }\int \int dydv_{a}\sigma_{0}(y,v_{a})\delta [y-(v_{s}-v_{a})/\Omega ].$$

Substituting into eq (19) and interchanging orders of integration, gives

$${\tilde{f}}_{0}(k,\Omega )=\int \int dydv_{a}\sigma_{0}(y,v_{a})\exp[-ik(\Omega y+v_{a})].$$ (24)

Now writing the two-dimensional Fourier transform of *σ*_0_*(y, v*_a_),

$${\tilde{\sigma }}_{0}(k_{y},k_{v})=\int \int dydv_{a}\sigma_{0}(y,v_{a})\cdot \exp[-i(k_{y}y+k_{v}v_{a})],$$

and comparing to eq (24), we see that

$${\tilde{f}}_{0}(k,\Omega )={\tilde{\sigma }}_{0}(k\Omega ,k).$$

This result resembles the central-slice theorem encountered earlier. Here ${\tilde{f}}_{0}(k,\Omega )$is equal to the two-dimensional Fourier transform, ${\tilde{\sigma }}_{0}(k_{y},k_{v})$, of *σ*_0_(*y*, *v*_a_) evaluated on a line in the (*k_y_, k_v_*) Fourier plane through the origin at angle tan^−1^(Ω) from the *k_v_*-axis. Thus, by varying both *k* and Ω, complete two-dimensional coverage of ${\tilde{\sigma }}_{0}(k_{y},k_{v})$ in Fourier space can be achieved. The reconstruction of *σ*_0_(*y*, *v*_a_) then follows on taking the inverse two-dimensional Fourier transform of ${\tilde{\sigma }}_{0}(k_{y},k_{v})$.

### Two-Dimensional Imaging (Tomography) with Unknown v_a_ Distribution

Here we wish to reconstruct the three-dimensional function *σ*_0_(*x′, y′, v*_a_,). This is the most general inversion problem considered. Now rewrite eq (15) as

$$\begin{array}{l} f_{t}(v_{s},\Omega )=\frac{1}{\Omega }\int \int \int dxdydv_{a}\sigma_{t}(x\prime ,y\prime ,v_{a})\\ \;\cdot \delta [y-(v_{s}-v_{a})/\Omega ]\\ \;=\frac{1}{\Omega }\int \int \int dx\prime dy\prime dv_{a}\sigma_{0}(x\prime ,y\prime ,v_{a})\\ \;\cdot \delta [-x\prime \sin\;\Omega t+y\prime \cos\;\Omega t-(v_{s}-v_{a})/\Omega ] \end{array}$$ (25)

using eqs (10) and (12b) in the last step. Substituting eq (25) into eq (19) then gives

$$\begin{array}{l} {\tilde{f}}_{t}(k,\Omega )=\int \int \int dx\prime dy\prime dv_{a}\sigma_{0}(x\prime ,y\prime ,v_{a})\\ \;\cdot \exp[ik\Omega (x\prime \sin\;\Omega t-y\prime \cos\;\Omega t-ikv_{a}]. \end{array}$$ (26)

Writing the three-dimensional Fourier transform of *σ*_0_(*x′*, *y′*, *v*_a_) as

$$\begin{array}{l} {\tilde{\sigma }}_{0}(k_{x},k_{y,}k_{v})=\int \int \int dx\prime dy\prime dv_{a}\sigma_{0}(x\prime y\prime v_{a})\\ \;\cdot \exp[-i(k_{x}x\prime +k_{y}y\prime +k_{v}v_{a})], \end{array}$$

and comparing to eq (26), we have

$${\tilde{f}}_{t}(k,\Omega )={\tilde{\sigma }}_{0}(-k\Omega \;\sin\;\Omega t,k\Omega \;\cos\;\Omega t,k).$$

This is the generalization of the central-slice theorem to three-dimensions. Thus, by suitably varying the three parameters *v*_s_, Ω and *t*, full coverage in the three-dimensional Fourier space can be achieved, and an inverse Fourier transform of ${\tilde{\sigma }}_{0}(k_{x},k_{y},k_{v})$ yields the reconstruction *σ*_0_(*x′,y′, v*_a_).

## 4. Spatial Resolution and the Image Point Spread Function

In previous sections, we considered the idealized case of an infinitesimally narrow linewidth *v_τ_*. This simplifies the mathematics, but implies that spatial resolution can be arbitrarily small. In this section, we show that spatial resolution is controlled by the natural linewidth *v_τ_*; in particular, spatial resolution is proportional to the ratio of *v_τ_* to the rotational velocity Ω of the absorber.

Below we derive the point spread function (PSF) of the two-dimensional tomographic reconstruction problem, with no *v*_a_ dependence for simplicity (the second case above). The PSF is by definition merely the reconstructed image of a two-dimensional Dirac delta function, or point object, and its width provides a reasonable measure of image resolution. Alternatively, the reconstructed image may be regarded as the true image convolved with the PSF.

To derive the point spread function in two dimensions, we repeat the above derivation using eq (14) in place of eq (15). Substituting

$$\sigma_{t}(x,y,v_{a})=\sigma_{t}(x,y)\delta (v_{a})$$

into eq (14) gives

$$f_{t}^{\left(\tau \right)}(v_{s},\Omega )=\int \int dxdy\sigma_{t}(x,y)P^{\left(\tau \right)}(\Omega y-v_{a}),$$

which is the first line of eq (18) with δ(·) replaced by *P*^(*τ*)^(·). Now write this as

$$\begin{array}{l} f_{t}^{\left(\tau \right)}(v_{s},\Omega )=\int \int dx\prime dy\prime \sigma_{0}(x\prime ,y\prime )\\ \;\cdot P^{\left(\tau \right)}[\Omega (-x\prime \sin\;\Omega t+y\prime \cos\;\Omega t)-v_{s}]. \end{array}$$ (27)

Writing the Fourier transform of $f_{t}^{\left(\tau \right)}(v_{s},\Omega )$with respect to *v*_s,_

$${\tilde{f}}_{t}^{\left(\tau \right)}\left(k,\Omega \right)=\int dv_{s}f_{t}^{\left(\tau \right)}\left(v_{s},\Omega \right)\exp\left(-ikv_{s}\right),$$ (28)

substituting eq (27) into (28) and interchanging orders of integration, results in

$$\begin{array}{l} {\tilde{f}}_{t}^{\left(\tau \right)}\left(k,\Omega \right)={\tilde{P}}^{\left(\tau \right)}\left(k\right)\int \int dx^{\prime }dy^{\prime }\sigma_{0}\left(x^{\prime },y^{\prime }\right)\\ \;\cdot \exp\left[ik\Omega \left(x^{\prime }\sin\;\Omega t-y^{\prime }\cos\;\Omega t\right)\right]\\ \;={\tilde{P}}^{\left(\tau \right)}\left(k\right){\tilde{\sigma }}_{0}\left(-k\Omega \;\sin\;\Omega t,k\Omega \;\cos\;\Omega t\right), \end{array}$$ (29)

where

$${\tilde{P}}^{\left(\tau \right)}\left(k\right)=\int dv_{s}P^{\left(\tau \right)}\left(-v_{s}\right)\exp\left(-ikv_{s}\right)$$ (30)

is the Fourier transform of *P*^(*τ*)^(−*v_s_*). Equation (29) is merely eq (22) multiplied by ${\tilde{P}}^{\left(\tau \right)}\left(k\right)$.

We can evaluate ${\tilde{P}}^{\left(\tau \right)}\left(k\right)$ by inserting eq (7) into (30); integrating, the result is

$${\tilde{P}}^{\left(\tau \right)}\left(k\right)=\exp\left(-kv_{\tau }\right).$$

Thus, the Fourier transform, ${\tilde{\sigma }}_{0}\left(k_{x},k_{y}\right)$, of σ_0_(*x′, y′*) is weighted by the exponential radially-dependent function exp(−*kv_τ_).* The point spread function is obtained by setting ${\bar{\sigma }}_{0}\left(k_{x},k_{y}\right)=1$ in eq (29); as a result, the PSF is just the two-dimensional inverse Fourier transform of ${\tilde{P}}^{\left(\tau \right)}\left(k\right)$. Since *k*Ω is the radial coordinate in the Fourier plane, ${\tilde{P}}^{\left(\tau \right)}\left(k\right)$ has radial symmetry and the resulting PSF also has radial symmetry. Hence, the inverse two-dimensional Fourier transform of ${\tilde{P}}^{\left(\tau \right)}\left(k\right)$ can be evaluated in polar form:

$$\begin{array}{l} \text{PSF}\left(r^{\prime }\right)=\frac{\Omega^{2}}{2\pi }\int_{0}^{\infty }dk\;k\;{\tilde{P}}^{\left(\tau \right)}\left(k\right)J_{0}\left(\Omega kr^{\prime }\right)\\ \;=\frac{\Omega^{2}}{2\pi }\int_{0}^{\infty }dk\;k\exp\left(-kv_{\tau }\right)J_{0}\left(\Omega kr^{\prime }\right)\\ \;=\frac{v_{\tau }}{2\pi }\frac{1}{{\left[{\left(r^{\prime }\right)}^{2}+{\left(v_{\tau }/\Omega \right)}^{2}\right]}^{3/2}}, \end{array}$$ (31)

where *J*_0_(·) is the zero-order Bessel function and $r^{\prime }=\sqrt{{x^{\prime }}^{2}+{y^{\prime }}^{2}}$. This is the desired point spread function whose width gives the image resolution. Let $r_{0}^{\prime }$ signify the full width at half maximum of eq (31); then

$$r_{0}^{\prime }=0.76\;v_{\tau }/\Omega .$$

The important thing to note is that $r_{0}^{\prime }$ is inversely proportional to the rotational velocity Ω. Finally, one can show that PSF(*r′*) becomes a two-dimensional Dirac delta function in the limit *v*_τ_→0, as required. As an example, for typical linewidths, *v_r_*, of less than a mm/s, this result shows that $r_{0}^{\prime }$ is on the order of a tenth of a millimeter when the absorber rotates at one revolution per second (Ω = 2π).

## 5. Imaging with an Extended Source (Non-Parallel Incidence)

In the latter two sections, we assumed parallel *γ*-ray incidence in the direction of the *x*-axis. Parallel incidence is easily achieved by placing the source far enough away so that it subtends a small angle at all points on the absorber. This is practical when the source is sufficiently strong to provide a *γ*-ray flux intercepting the absorber that results in an acceptable signal-to-noise ratio. Unfortunately, arbitrarily strong Mössbauer sources are not available, so that moving the source close to the absorber to maximize the incident flux may be important.

**Figure 4:**
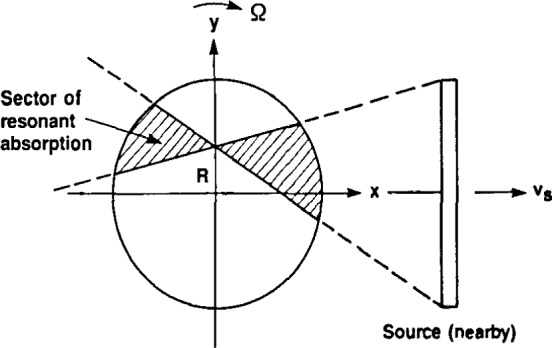
For an extended source (non-parallel *γ*-ray incidence), the line of sensitivity broadens into a sector of sensitivity bounded by lines subtending the source and intersecting at the point (*x*=0, *y*=*R*), where *R*Ω=*v*_1_−*v*_a_.

**Figure 5:**
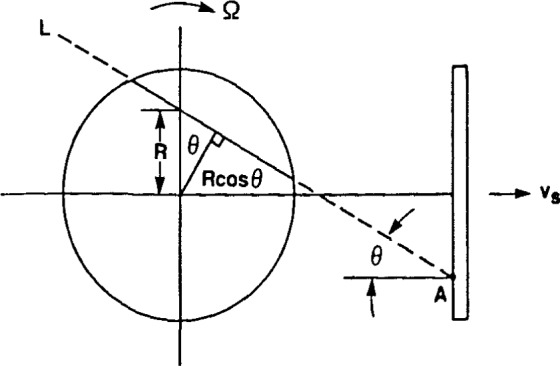
The line *L* signifies the path of a *γ*-ray emitted from one point on the extended source.

In the latter case, the source must be regarded as spatially extended, say, in the *y*-direction (fig. 4), and the incident *γ*-rays are no longer parallel. Assume that the extended source is moving in the *x*- direction at velocity *v*_s_ and that the absorber rotates with angular velocity Ω as before. Also, for simplicity, assume that the intrinsic velocity *v*_a_=0. In this geometry, one can show that the component of velocity of a point on the rotating absorber and of a point on the source in the direction of the line joining them (i.e., along the line of flight of a *γ*-ray passing between the two points) are equal to each other (the resonance condition) if and only if the line defined by the two points passes through (*x*=0, *y=v*_s_/Ω) in the stationary (*x, y*) coordinate system. To see this, refer to the line *L* in figure 5, which we suppose is the path of a *γ*-ray emitted from point A on the source. Suppose this path intersects the *y*-axis at height *R*, as shown. Then *L* is tangent to a circle around the axis of rotation of radius *R* cos *θ*, where *θ* is the angle between *L* and the *x*-axis. From previous arguments, all points in the absorber lying on *L* have the same component of velocity in the direction of *L*, namely Ω*R* cos *θ.* But the additional component of velocity of the incident *γ*-ray along *L* due to the motion of the source is *v*_s_ cos *θ*, as can be seen from figure 5. The resonance condition is then obtained by equating the absorber and source components of velocity along *L*; thus setting Ω*R* cos *θ=v*_s_ cos *θ* results in *R*=*v*_s_/Ω. Consequently, only those *γ*-rays moving along paths through (*x*=0, *y*=*v*_s_/Ω) are resonantly absorbed.

**Figure 6:**
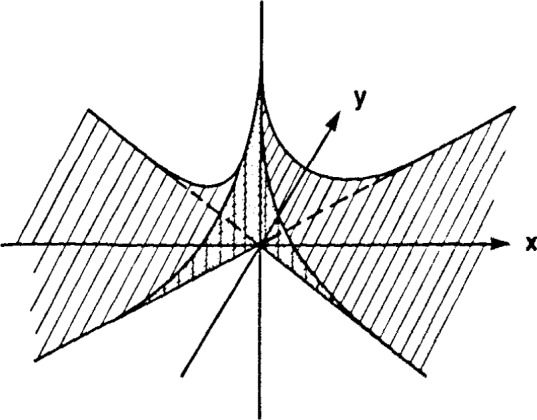
Probability distribution of resonant absorption per unit area over the sector of sensitivity in figure 4. The distribution falls off as 1/|*x*| along the *x*-axis and is approximately of width *v*_τ_/Ω along the *y*-axis.

The complete set of lines passing through this point and intercepting the source generates a sector, as shown in figure 4, where the boundaries of the sector are the two lines intersecting the end points of the extended source and coming together at the point (0, *v*_s_/Ω) in the (*x, y*) system. The density of lines peaks at the point (0, *v*_s_/Ω) and falls off in proportion to 1/|*x*| in the *x*-direction (fig. 6). Through the peak in the *y*-direction, the width of this function is much narrower; in particular, the width in the *y*-direction can be shown to be *v*_τ_/Ω, which is approximately the width of the point spread function derived previously for parallel incidence. Neglecting, for the moment, attenuation of the *γ*-rays through the absorber, the line-density function sketched in figure 6 may be equivalently interpreted as the probability density function of resonant absorption events per unit area, which is just proportional to the inverse of the sector area shown in figure 4.

The highly peaked distribution of the probability of resonance events is significant, since it implies that merely by varying *v*_s_ and counting absorption events as a function of time, it should be possible to generate a blurred image even without image reconstruction. Figure 4 shows that, by rotating the absorber, the *γ*-rays are effectively “focused” through the point (0, *v*_s_/Ω). This focusing-like effect can be used to create a blurred image without further processing. A complete or deblurred image reconstruction cannot in this case be carried out analytically, but the reconstruction nevertheless can be performed using iterative algebraic reconstruction techniques (ART) [4,5]. The ART algorithms assume an initial estimate of the unknown image and then, on the basis of this estimate, recompute the measurements (line integrals of the resonant absorption coefficient). The recomputed measurements are then subtracted from the true measurements, giving rise to a set of error terms, which are then used to systematically adjust the current image estimate to reduce this error. When this iterative procedure is continued, it can be shown that the overall error tends to zero and, in the absence of measurement uncertainty and incomplete data, the reconstructed image converges to the true image. Such iterative approaches also make it relatively easy to incorporate attenuation effects in the algorithm.

In the above discussion, we assumed for simplicity that the extended source was one-dimensional (i.e., extended along the *y*-direction). If the source has some depth as well as height (i.e., some extension in the *z*-direction), the additional degree of freedom arising from variations in the *x*-component of the *γ*-ray velocity will broaden somewhat the region of sensitivity beyond the sector illustrated in figure 4. To minimize this broadening effect, the source should be collimated in the *z*-direction as much as practical with a suitable arrangement of slits in this direction. A three-dimensional absorber could then be reconstructed on a “slice-by-slice” basis, as in two-dimensional *x*-ray tomography.

## 6. Applications

Applications of the technique to interior imaging appear to be limited to lighter materials, or absorbers of small size, that permit sufficient *γ*-ray penetration. One possibility is the imaging of composite materials, in which a Mössbauer element is embedded in a lighter matrix. Another possibility is surface imaging, whereby the surface of a flat absorber is rotated about an axis normal to the surface and a one-dimensional extended source is displaced a small amount from the plane of the surface. If all the impinging *γ*-rays have the same (or nearly the same) *z*-component of velocity, the region of sensitivity in which resonant absorption takes place will look much the same as the sector illustrated in figure 4. In this case, the value of *v*_s_, used previously should be replaced by the component of the source velocity in the plane of the surface.

Some potential high-resolution applications in materials science include the imaging of grain boundary segregation, the imaging of residual stress distributions, and the imaging of the distribution of magnetism in ferromagnetic materials due to perturbations in the Mössbauer spectra arising from variations in the magnetic hyperfine field.

## 7. Conclusion

In conventional Mössbauer spectroscopy, the measurement is spatially integrated over the absorber. For parallel *γ*-ray incidence, a rotating absorber generates a line of “sensitivity” over which resonant absorption takes place, where the location of the line is a function of the source velocity and the absorber rotational velocity. In particular, this line is parallel to the velocity of the source and passes through the point (*x*=0, *y*=*v*_s_/Ω). For an extended source, the line broadens into a sector subtending the source and coming together at the point (*x*=0, *y*=*v*_s_/Ω). For a highly extended source, the result is as if the *γ*-rays were effectively “focused” through the point (*x*=0, *y=v*_s_*/*Ω). This focusing effect can be used to generate a blurred image of the absorption coefficient distribution in the absence of further processing when the measurements are recorded as a function of *v*_s_ and time (or the instantaneous absorber rotational position). Furthermore, by exploiting the three measurement degrees of freedom, *v*_s_, Ω, and time (or rotational position), complete spectroscopic information can, in principle, be recovered as a function of position. Thus, Mössbauer spectroscopy can be performed in an imaging mode. Spatial resolution is proportional to the ratio of the natural linewidth *v_τ_* to the rotational velocity Ω, but becomes rapidly signal-to-noise limited as the resolution increases.

## Footnotes

1 Figures in brackets indicate literature references.
